# Retraction Note: The teratogenic effect of pregabalin on heart, liver and kidney in rats: a light microscopic, electron microscopic and immunohistochemical study

**DOI:** 10.1186/s40360-023-00697-4

**Published:** 2023-10-13

**Authors:** Omnia I. Ismail, Eman S. Shaltout, Nora Z. Abdellah, Diab F. Hetta, Wael M. A. Abd El-Ghani, Lobna A. Abdelzaher, Ahmed Mohamed Mohamed Mahmoud, Asmaa M. Hasan, Noha A. Rashed, Noha Esmael Ebrahem

**Affiliations:** 1 Department of Human Anatomy and Embryology, Faculty of Medicine, Assiut University, Assiut, 71515 Egypt; 2 Department of Forensic Medicine & Clinical Toxicology, Faculty of Medicine, Assiut University, Assiut, 71515 Egypt; 3 Department of Anesthesia and Pain Management, South East Institute, Assiut University, Assiut, 71515 Egypt; 4 Department of Neurosurgery, Faculty of Medicine, Assiut University, Assiut, 71515 Egypt; 5 Department of Pharmacology, Faculty of Medicine, Assiut University, Assiut, 71515 Egypt; 6 Department of Neuropsychiatry, Assiut University hospital, Assiut, 71515 Egypt

**Retraction Note: BMC Pharmacology and Toxicology (2022) 23:4**

10.1186/s40360-021-00546-2

The Editor has retracted this article. After publication, concerns were raised regarding image similarities and irregular features in the presented figures. Specifically:

Fig. 2d appears to contain highly similar areas, and an unusual diagonal artifact that appears to duplicate nearby features of the image.

Fig. 2e appears to contain highly similar areas within the image.

Fig. 3a and b appear to overlap.

Fig. 5a and b appear highly similar, with some minor differences.

Fig. 8b appears to contain highly similar areas within image.

Fig. 10a appears to contain unusual features in the image.

The Editor therefore no longer has confidence in the presented data.

Noha Esmael Ebrahem, Eman S. Shaltout, Wael M. A. Abd El-Ghani, Noha A. Rashed, Asmaa M. Hasan, Omnia I. Ismail, Ahmed Mohamed Mohamed Mahmoud and Nora Z. Abdellah do not agree to this retraction. Diab F. Hetta and Lobna A. Abdelzaher has not responded to any correspondence from the editor or publisher about this retraction.

